# In Vitro Models of Head and Neck Cancer: From Primitive to Most Advanced

**DOI:** 10.3390/jpm13111575

**Published:** 2023-11-03

**Authors:** Irina Arutyunyan, Enar Jumaniyazova, Andrey Makarov, Timur Fatkhudinov

**Affiliations:** 1 Research Institute of Molecular and Cellular Medicine, RUDN University, 6 Miklukho-Maklaya Street, 117198 Moscow, Russia; labrosta@yandex.ru (I.A.); anvitmak@yandex.ru (A.M.); tfat@yandex.ru (T.F.); 2 National Medical Research Center for Obstetrics, Gynecology and Perinatology Named after Academician V.I. Kulakov Ministry of Healthcare of the Russian Federation, 4 Oparina Street, 117997 Moscow, Russia; 3 Histology Department, Pirogov Russian National Research Medical University, Ministry of Healthcare of the Russian Federation, 117997 Moscow, Russia; 4 Avtsyn Research Institute of Human Morphology of Federal State Budgetary Scientific Institution Petrovsky National Research Centre of Surgery, 3 Tsyurupy Street, 117418 Moscow, Russia

**Keywords:** head and neck squamous cell carcinoma, in vitro models, 3D models, viral infection, personalized medicine

## Abstract

For several decades now, researchers have been trying to answer the demand of clinical oncologists to create an ideal preclinical model of head and neck squamous cell carcinoma (HNSCC) that is accessible, reproducible, and relevant. Over the past years, the development of cellular technologies has naturally allowed us to move from primitive short-lived primary 2D cell cultures to complex patient-derived 3D models that reproduce the cellular composition, architecture, mutational, or viral load of native tumor tissue. Depending on the tasks and capabilities, a scientific laboratory can choose from several types of models: primary cell cultures, immortalized cell lines, spheroids or heterospheroids, tissue engineering models, bioprinted models, organoids, tumor explants, and histocultures. HNSCC in vitro models make it possible to screen agents with potential antitumor activity, study the contribution of the tumor microenvironment to its progression and metastasis, determine the prognostic significance of individual biomarkers (including using genetic engineering methods), study the effect of viral infection on the pathogenesis of the disease, and adjust treatment tactics for a specific patient or groups of patients. Promising experimental results have created a scientific basis for the registration of several clinical studies using HNSCC in vitro models.

## 1. Introduction

As of 2019, cancer is the leading cause of death for the age of 30–70 in 57 countries, including USA, Canada, Australia, China, and most European countries. It is expected to move to first place in the ranking in other developed and developing countries, which is now taken by cardiovascular diseases [1], and the projected annual number of deaths from cancer will exceed 16 million by 2040 [2]. An increase in cancer survival rates witnessed in recent years is largely due to improved diagnostics rather than breakthroughs in treatment effectiveness, despite significant advances in the field of anticancer drug therapy [3].

The success of early diagnosis and therapy varies significantly depending on cancer type [4]. Head and neck squamous cell carcinoma (HNSCC), one of the ten most common cancers, mostly arises from mucosal epithelium in the oral cavity, pharynx, and larynx in close proximity to a number of vital structures, which complicates the radical surgical intervention. This tumor is characterized by late diagnosis, rapid progression, and resistance to antitumor treatment strategies [5,6].

Evidence-based prevention strategies for HNSCC encompass various approaches, including lifestyle modifications, vaccination against viral infections, and improvement of detection methods. Detection of HNSCC in the early stages significantly increases the patient’s chances of a successful outcome: the 5-year survival is 86.6% for localized disease (stages I and II), 69.1% for locally advanced disease (stages III-IVB), and 39.3% for metastatic disease (stage IVC). As examples of diagnostic advancements, we can cite the development of the method of robotic surgery for the detection of the primary tumor site in patients with head and neck unknown primary cancers, mass screening for human papillomavirus or Epstein–Barr virus, and implementation of careful physical examination of the oral cavity and fiber-optic nasopharyngolaryngoscopy as components of mandatory medical examination of high-risk patients [5,6].

HNSCCs are variably sensitive to radiation and drug treatment, while the locally advanced or metastastatic cases require a combination of surgery, radiation therapy, and chemotherapy [6]. However, even with the use of several types of antitumor treatment, the recurrence rate of HNSCC reaches 50% [7]. Drug resistance and toxicity limit the effectiveness of chemotherapy drugs used to treat HNSCC, such as cis- or carboplatin, 5-fluorouracil, and taxanes [8,9]. The introduction of new targeted agents (cetuximab) and immunotherapy drugs (nivolumab, pembrolizumab) improved the clinical outcomes, albeit without solving the problem of treatment resistance in the majority of patients with HNSCC [10,11].

The scarcity of biomarkers of HNSCC response to anticancer therapy reflects the lack of relevant prognostic models for HNSCC [12,13,14]. Robust preclinical models are required to address the mechanisms of treatment resistance and progression of HNSCC in order to update the therapeutic strategies [15,16].

## 2. Transition from 2D to 3D HNSCC In Vitro Models

In the middle decades of the 20th century, scientists used cutting-edge cell technologies in order to provide convenient and usable systems for laboratory investigation of the biological properties of tumor cells. For the first time, primary cell cultures from clinical material of patients with HNSCC were successfully isolated almost half a century ago [17]. Although the main emphasis was initially placed on isolating the population of tumor cells, later studies demonstrated the significance of the interaction between tumor and tumor-associated stromal cells during co-cultivation [18]. Many tumor cultures have been immortalized; to date, several hundred such HNSCC lines have been obtained [15,19,20,21]. Among the 39 most commonly used HNSCC cell lines in research, the majority (about 60%) are derived from oral tissue, since the main treatment for this cancer is surgery, which allows researchers to provide enough material for cell isolation. Cell lines derived from laryngeal tumors account for 15% of the total, 10% are isolated from the pharynx, less than 3% from the nasal septum, and the origin of another 12% is uncertain [22]. In another study, when analyzing all 383 HNSCC lines known to the authors at that time, a slightly different distribution was revealed: 45% (171 lines) were obtained from oral cavity tumors, 27% (104 lines) from the larynx, 14% (55 lines) from the oropharynx, 10% (37 lines) from the hypopharynx, 2% (9 lines) from facial skin, and 2% (7 cell lines) from the paranasal/nasal sinus [21].

Surprisingly, the list of 73 (60 main and 13 additional) human tumor cell lines used in The NCI-60 Human Tumor Cell Lines Screen, designed to identify and characterize novel compounds with growth inhibition or killing of tumor cells, does not include a single HNSCC line [23]. To study this tumor type, a global biological resource, the American Type Culture Collection (ATCC), offers a panel of six cell lines (Table 1) [24,25].

As can be seen from Table 1, all presented lines carry mutations in one (TP53) or several genes. In the work of Li et al. the genomic data of 39 HNSCC cell lines were compared with the genomic findings in 106 HNSCC tumors. TP53 was the most commonly mutated gene both in HNSCC tumors and cell lines. Amplification of eight genes (PIK3CA, EGFR, CCND2, KDM5A, ERBB2, PMS1, FGFR1, and WHSCIL1) and deletion of five genes (CDKN2A, SMAD4, NOTCH2, NRAS, and TRIM33) were found in both HNSCC cell lines and tumors; all of these genes had higher alteration frequencies in HNSCC cell lines compared with primary tumors. These findings suggest that mutations and gene copy-number alterations that exist in HNSCC cell lines may result from selection and/or propagation in cell culture that does not reflect critical biological properties of this cancer. Moreover, seventeen genes were only mutated in HNSCC cell lines, but not in human tumors, suggesting that these mutations may arise through immortalization in tissue culture [22].

It is obvious that 2D HNSCC cell lines have significant phenotypic, physiological, genetic, and epigenetic differences compared to the tumor tissue from which they were obtained, due to clonal selection induced by selective survival pressures intrinsic to the culture conditions [22,24,26,27,28]. There is another trap in working with immortalized lines: hundreds of them are misidentified. The most obvious example is the Hep-2 line, first described in 1954 as laryngeal cancer cells. This line is also still actively used for in vitro studies of HNSCC, although back in 1966 it was determined that this line (like many others) was contaminated and then completely replaced by human cervical adenocarcinoma HeLa cells [29,30].

For several decades, preclinical studies of HNSCC have been carried out on in vitro models, which make it possible to evaluate the effectiveness of antitumor therapy, study resistance mechanisms, and improve early diagnostic and screening tests [15,16,31,32]. The advantages of cell lines as the most simplified in vitro tumor model are clear: ease of use, precise control of exogenous factors, economic accessibility, and wide range of applications [16,18].

At the same time, the main problem is their inconsistency with the 3D architecture of tumor tissue, which provides a gradient of gases, nutrients, and biologically active substances that can be reproduced in in vivo models [32,33,34,35]. To overcome this discrepancy, immortalized cells can be transplanted subcutaneously or organotopically into immunodeficient animals, obtaining cell-line derived xenografts, which are voluminous neoplasms, often capable of metastasis [15].

The possibility of obtaining such xenografts has been shown for many HNSCC cell lines (for example, A-253 [36,37], FaDu [37,38], CAL27 [39], and Detroit 562 [40]) and is actively used to evaluate the effectiveness of antitumor agents, search for methods for imaging tumor cells in vivo, or study the mechanisms of carcinogenesis. However, this path of transition to a 3D model of HNSCC cannot be called optimal, since immunodeficient animals require enormous financial and labor costs, obtaining a tumor volume sufficient for research work (150–300 mm^3^) takes several weeks, and some of the planned studies may turn out to be completely unavailable, for example, due to high radiosensitivity of some strains of mice (NOD-SCID), which precludes experiments using radiation therapy [33,41]. Moreover, not all HNSCC cell lines are tumorigenic; among the ten lines first described in 1981, only three have been confirmed to grow as transplantable xenografts [17].

An attempt to find a balance between the availability and reproducibility of simple models based on 2D adherent tumor lines and the relevance of labor-intensive 3D in vivo animal-based models ultimately led researchers to the creation of 3D in vitro cell models, the variety of forms of which allows studying different aspects of tumor tissue biology [16,42,43]. It is important to note that the development of 3D cell models, and their improvement and implementation in preclinical practice, contributed to the rationalization of the use of animals in research, complying with the principles of “The Three R’s” (Reduction, Replacement, and Refinement) in animal research [44,45].

## 3. Three-Dimensional In Vitro Cell Models of HNSCC: Main Types and Methods of Production

### 3.1. Spheroids

Multicellular spheroids collected from tumor cells are considered the most simple and reproducible in vitro tumor model capable of reflecting the response of tumor tissue to therapy [46,47,48,49]. Changing cultivation conditions during the transition from 2D to 3D leads to the formation of volumetric multicellular conglomerates, in which cells interact with each other and the external environment without the participation of a culture substrate; as a result, pathophysiological gradients characteristic of tumor tissue are formed in the spheroids [50,51,52,53]. Spheroids are characterized by cellular zoning, which begins to clearly manifest itself as the size of the spheroid increases; upon reaching a diameter of 500 μm, a hypoxic zone and foci of necrosis are formed in the spheroid. Histological examination allows the identification of several layers in large spheroids: an outer layer represented by rapidly proliferating cells, a middle layer with senescent or quiescent cells, and an inner layer containing necrotic cells [46,53,54,55]. These heterogeneous layers are the result of limited diffusion of oxygen and nutrients into the multicellular structure. Cells in the outer layer multiply rapidly due to easier access to oxygen, nutrients, and growth factors; closer to the center of the spheroid, the supply of oxygen and nutrients decreases, and the amount of carbon dioxide and decay products increases [50,53,56].

Large spheroids with a diameter of 500 μm or more reproduce the oxygen gradient characteristic of tumor tissue, forming a focus of hypoxia in its center, which affects the processes of tumorigenesis, activating protumor transcription [57,58] and triggering mechanisms for the development of chemo- and radio-resistance, which lead to increased tumor growth and worsening patient outcomes [59,60,61]. Under conditions of insufficient tissue perfusion, protons and lactic acid accumulate in the extracellular space, leading to the formation of an acidic extracellular microenvironment and creating a pH gradient. This phenomenon is similar to the Warburg effect associated with lactate accumulation in solid tumors [62].

The formation of spheroids occurs spontaneously under conditions where cell–cell interactions predominate over interactions between cells and the culture substrate. In fact, all the main methods for forming spheroids are aimed at creating the following conditions: the hanging drop method, culturing cells in dishes or vials with an ultra-low adhesion surface, and culturing using rotational systems or magnetic fields [46,63,64,65]. In each of these methods, the main task is to minimize the contact of cells and the culture substrate; however, the methods for solving this problem differ:

(1) completely remove the substrate, culturing cells in a drop supported by surface tension (hanging drop method) [46,66,67];
(2) modify the substrate, eliminating the possibility of cells to attach to it (culturing in dishes or plates with an ultra-low adhesion surface) [68,69,70];
(3) ensure continuous mixing of the cell suspension, preventing cells from settling and coming into contact with the substrate (agitation-based method, magnetic levitation) [53].

The first two methods are most commonly used to create spheroids from immortalized cell lines or primary cell cultures isolated from tumor tissue of patients with HNSCC [67,70]. Primary cultures are inherently heterogeneous and are obviously more relevant models for use in personalized medicine. However, the process of obtaining primary cultures from HNSCC biopsies does not always lead to the desired result:

(1) part of the material may be initially contaminated with bacteria or fungi;
(2) during the cultivation process, epithelial cells can be replaced by more rapidly proliferating stromal cells;
(3) the rate of cell proliferation and the efficiency of spheroid formation decreases with an increase in the number of passages performed;
(4) some methods for obtaining spheroids from such cultures are ineffective [71,72].

To reproduce the heterogeneous composition of tumor tissue, it was proposed to use heterospheroids (or heterotypic spheroids, co-culture spheroids) [73], consisting of a well-characterized immortalized line and an additional cellular component usually associated with tumor cells in vivo, for example, immune cells, fibroblasts, or endothelial cells. Currently, there is active research work using heterospheroids; however, in most cases they are used to model liver and pancreatic tumors, and only a few studies have been performed with heterospheroids based on HNSCC tumor lines (Table 2).

Thus, reproducible and accessible protocols for obtaining spheroids from immortalized lines or primary cultures of HNSCC allow the creation of in vitro models, in which diffusion restrictions are reproduced not only for oxygen and nutrients, but also for the transport of biologically active substances, imitating the physiological barriers existing in vivo. In many respects, spheroids are closer to tumor tissue than 2D cell cultures, which allows them to be used as a relevant preclinical model for testing the efficacy and toxicity of many anticancer drugs [46,50,66,68,78,79].

### 3.2. Tissue-Engineered Models

The 2D cultures, spheroids, and heterospheroids described above have a significant drawback as an in vitro model, namely the lack of interaction between tumor cells and the extracellular matrix (ECM), which plays an important role in the occurrence and progression mechanisms of HNSCC [80]. In order to increase the relevance of in vitro tumor models, tissue engineering methods were used, namely cell culture on 3D scaffolds. The first attempts to cultivate HNSCC cells in collagen gel were made at the end of the 20th century [81,82], and the first model of HNSCC using a synthetic scaffold was created in 2007, based on oral squamous cell carcinoma (OSCC-3) cells cultured within a highly porous poly(lactide-co-glycolide) scaffold [83]. Currently, type I collagen in the form of a gel [84] or a porous matrix [85] is most often used as a scaffold material for modeling HNSCC. Such scaffolds are populated with both immortalized tumor cells (SCC090, UM-SCC6 [85], FaDu [86]), and primary cultures obtained from tumor tissue of patients with HNSCC [84,85].

A more complex technology for creating a 3D model of tumor tissue using TRACER (the Tissue Roll for Analysis of Cellular Environment and Response) is also described. It is a novel model in which cells (HNSCC cell lines CAL33 or FaDu co-cultured with cancer-associated fibroblasts) are embedded in a collagen hydrogel infiltrate into a porous cellulose scaffold that is then rolled around an aluminum core to generate a multi-layered 3D tissue [87,88].

Such models are very convenient for studying the interaction of tumor cells and individual proteins of the extracellular matrix or stromal cells [88], and the variability of the scaffolds used makes it possible to control the conditions of this interaction, for example, providing normoxia [87] or hypoxia [85]. By analogy with already developed tissue engineering models of other types of solid tumors, it is, in principle, possible to model HNSCC using a wide variety of scaffolds based on biocompatible natural or synthetic polymeric materials and their composites [89,90,91,92].

Another option for this type of in vitro model is the cultivation of tumor cells on decellularized ECM, when cellular components are removed, and the biological composition is mostly preserved. A study by He et al. compared the proteome of normal and tumor decellularized ECM from oral cavity tissues. It was found that 26 proteins only showed in tumor ECM, 14 proteins only showed in late-stage tumor ECM, and most variant proteins were linked to metabolic regulation and tumor immunity. Tumor ECM influenced the proliferation, apoptosis, and migration of tumor cells, as well as polarized macrophages towards an anti-inflammatory phenotype, which once again confirms the hypothesis of a significant influence of the ECM on the progression of HNSCC [80].

Thus, tissue-engineered in vitro tumor models provide a native-like tissue context for studies of HNSCC biology, but their applications are still at an early stage.

### 3.3. Bioprinted Models

In recent years, the method of 3D printing of tissues (including tumor tissues) has been actively developing. This method makes it possible to create complex, volumetrically defined structures, which are also anatomically accurate and relevant [93]. The method consists of constructing 3D structures by precise spatial superimposing layers of bioink that contain cells or cell conglomerates, cytokines, and extracellular matrix components [94,95]. Bioink is usually biocompatible hydrogel loaded with single cells or cell spheroids [96].

Three-dimensional printing was developed several decades ago, but only recently has this versatile technology been adapted to the biomedical field for the fabrication of complex structures using biocompatible materials [94,97]. Inkjet printing [98], extrusion printing [99], laser printing [100], and stereolithography [101] are used for 3D tissue bioprinting. These methods differ from each other in many respects: the cost of equipment and consumables, printing speed, resolution, restrictions on the maximum linear dimensions of the created object, the set of biogels suitable for work, etc. All these bioprinting methods enable a highly improved control of cell distribution within the 3D space compared to conventional approaches [94,102]. The created 3D models are structurally stable over a wide temperature range of 4 °C to 37 °C, which is a necessary condition for working with biological objects [93].

Currently, there are not many works describing the successful modeling of HNSCC using the bioprinting method. In a study by Kort-Mascort et al., emphasis was placed on the development of a new printable biogel containing alginate and gelatin as rheological modifiers, which impart mechanical integrity to the biologically active decellularized ECM (dECM), derived from porcine tongue after its decellularization and solubilization. The topographical characterization of the bioink showed a fibrous network with nanometer-sized pores. Immortalized cell lines UM-SCC-12 (larynx carcinoma) or UM-SCC-38 (oropharynx carcinoma) were selected as the cellular component. The model in the shape of discs with a 5 mm diameter and 500 µm height was printed using extrusion printing. The model was highly reproducible and allows proliferation and reorganization of HNSCC cells while maintaining cell viability above 90% for periods of nearly 3 weeks; cells produced spheroids having a cross-sectional area of at least 3000 μm^2^ by day 15 of culture and were positive for cytokeratin. The resulting 3D model was used to assess the response of tumor cells to cisplatin and 5-fluorouracil [103]. The authors continued their work in this direction and recently published data on an improved 3D model of HNSCC, in which the cellular component was represented by two types of cells—UM-SCC-38 and A8-HVFFs (immortalized human vocal fold fibroblasts) in a ratio of 1:2. In the process of culturing the model, spheroid development and growth over time with cancer cells in the core and fibroblasts in the periphery were observed [104].

It can be concluded that the 3D bioprinted model of HNSCC is essentially an improved tissue-engineered model, where a hydrogel is used as a scaffold, and the use of a printing method instead of the usual layering of the gel on a substrate allows for more accurate control of the compliance of the tissue architecture with the specified parameters. So far, bioprinted HNSCC models have been obtained only for immortalized lines, which is due to the need to use a large number of cells to obtain the desired result (in [104] cells were encapsulated in gel at a final concentration of 10 million cells per ml). Obviously, an important task for researchers is to obtain such 3D models with HNSCC patient-derived cells to closely match more biological features and potentially use this technique for personalized medicine.

### 3.4. Organoids

The technology of obtaining organoids, which represent an improved in vitro model that combines the advantages of spheroids (the ability of cells to 3D self-organization) and tissue engineering models (the presence of ECM) helped researchers to advance even further on the path to studying the interaction of tumor cells and the ECM [105]. Based on grammar, “-oid” is a suffix meaning “resembling”; organoid thus means “resembling an organ”, so organoids have to exhibit at least some organ functionalities of the modeled tissue [106]. Over the years of improving protocols for the production and use of organoids as an in vitro model of tumor tissue (in this case, the term “tumoroid” is also used), their main mandatory properties have been determined:

(1) organoids contain tumor cells at different levels of differentiation, including cancer stem cells;
(2) organoids consist of several cell types that self-organize in space, reproducing the architectonics of the original tumor tissue;
(3) self-organization of the organoid occurs in the presence of the ECM;
(4) the interaction of cells and the ECM allows reproduction of the functional properties of the simulated tumor tissue [106,107,108].

Thus, organoids have high genetic and phenotypic similarity to native tissue, maintaining the original intratumoral heterogeneity [109,110].

Most often, organoids for modeling HNSCC are obtained from resected tumor tissue of the patient. In the first stage, the biopsy sample is cleared of tissues of the peritumoral area (connective, muscle, fat), then washed very thoroughly, since the area of material collection (oral cavity, pharynx, nasal septum) is highly likely to be contaminated with bacteria or fungi. Next, the tissue is subjected to mechanical and enzymatic disaggregation using collagenases, dispase, hyaluronidase, trypsin, and EDTA (separately or as part of a cocktail of enzymes), after which sieved single cells or cell clusters are plated in a 3D extracellular matrix (ECM) hydrogel such as basement membrane extract (BME), where they form complex conglomerates [111]. This matrix is obtained from the basement membrane of sarcoma of Engelbreth-Holm-Swarm (EHS) mice, and is rich in such ECM proteins as laminin (a major component), collagen IV, heparan sulfate proteoglycans, entactin/nidogen, and a number of growth factors. It is easy to use and commercially available in standard, protein-enriched, or growth-factor reduced versions [112]. On the other hand, the EHS matrix is extremely complex; proteomic analysis shows that it contains more than 1800 unique proteins, the ratio of which varies from lot to lot, which can affect the reproducibility of the properties of organoids grown in it. In addition, the matrix obtained from mouse tumor tissue has an obvious drawback—it does not meet animal-derived-free standards, which will undoubtedly become mandatory for biomedical products in the near future, so researchers are actively looking for a replacement among synthetic or natural hydrogels [113,114].

Over the past few years, organoids have been successfully obtained from normal and tumor tissues of the brain, lungs, esophagus, stomach, intestines, liver, pancreas, kidneys, salivary glands, ovaries, mammary glands, prostate, and other organs [115]. For each source, a lengthy selection of optimal conditions for the formation of organoids was necessary. In protocols for obtaining organoids from head and neck tumor tissue, the steps for isolating individual cells or clusters do not differ fundamentally between different research groups, but the composition of the culture medium varies significantly (Table 3).

The number of studies that resulted in the successful production of HNSCC organoids is small due to difficult logistics (the time from resection of tumor tissue to delivery to the laboratory must be minimized), an often insufficient volume of biomaterial, as well as high requirements for the qualitative and quantitative composition of the culture medium, which demands the mandatory presence of growth factors, cytokines, and inhibitors of signaling pathways leading to the triggering of apoptosis or epithelial-mesenchymal transition (Table 3). At the same time, the efficiency of obtaining organoids from tumor tissue of the head and neck does not exceed 60–70%, even in laboratories that have been actively working with this material for many years (Table 3).

It is believed that the main factor influencing the efficiency of obtaining this 3D model of HNSCC is the quality of the initial biomaterial, since necrotic tumor tissue has poor organoid-forming efficacy. To minimize cell death and increase organoid outgrowth, Driehuis et al. recommend to transport tumor pieces in ice-cold basic medium containing 10 μM Rho kinase (ROCK) inhibitor compound Y-2763 or (less desired) ice-cold PBS [111]. It has also been shown that organoids can be established from cryopreserved tissues, although the efficacy of derivation is probably lower than that obtained when starting with fresh material [111]. According to Wang et al., the efficiency of obtaining HNSCC organoids is also affected by:

(1) mass of biomaterial (for tumor samples weighing less than 50 mg, the efficiency of obtaining organoids from it is reduced to 3%);
(2) time of transportation of tumor tissue to the laboratory (time exceeding 24 h leads to a decrease in efficiency from 60–70% to 22%);
(3) composition of the culture medium (for example, concentration of Wnt3a, R-spondin-1, EGF, Y27632, Noggin, and FGF2);
(4) ECM used (Matrigel is preferable to collagen I);
(5) primary/recurrent tumor status (for nasopharyngeal carcinoma, the efficiency of obtaining organoids is 82% for recurrent tumor and only 47% for primary tumor) [116].

In the same study, Wang et al. showed that the effectiveness of obtaining organoids from nasopharyngeal carcinoma tissue does not depend on the gender and age of the patient, or on the clinical stage or lesion location (primary focus or metastasis) of the tumor [116].

Thus, obtaining organoids requires significant material and time, as well as highly qualified personnel and close coordination of clinicians and cell biologists. The result is a tumor model that can be maintained in vitro for a long time, while maintaining genetic stability [118,119] and fairly fully reproducing the morphological characteristics of the original tissue. For example, organoids from nasopharyngeal carcinoma do not express CK7, unlike organoids from normal mucosa obtained from the same patient, which corresponds to differential expression of this marker in the original tissues [116]. It has been shown that organoids, when cultivated, retain the expression of many other markers of head and neck tumors, including those used to identify cancer stem cells: CK13, CK18, ALDH1A1, BMI-1, CD44, and CD133 [116,117,119,120,121]. Interestingly, lactate, which is promoted by Wnt activity, is required to maintain the population of CD133+ cells in HNSCC organoids. Moreover, silencing monocarboxylate transporter 1 (MCT1), the prominent pathway for lactate uptake in human tumors, with siRNA significantly impaired organoid-forming capacity of oral squamous cell carcinoma cells, which allows MCT1 to be considered as a potential therapeutic target for the treatment of HNSCC [121].

Organoids are considered highly relevant in vitro tumor models because they are able to preserve a unique set of biomarkers from donor tissue [122,123]. Increasing evidence indicates that organoids can predict response to treatment of the tumor from which they are derived [124]. In a pilot study, organoids obtained from 21 patients with gastrointestinal tumors predicted response to various types of chemotherapy with 100% sensitivity and 93% specificity [125]. A number of studies have highlighted the prognostic value of organoids in predicting the response of solid tumors to radiotherapy and its combination with other treatments [126,127]. In addition, organoids can also be expanded in vitro and cryopreserved, facilitating the establishment of an organoid biobank of different cancer subtypes from a large number of patients, representing an extremely useful tool for preclinical research [17,54,128].

### 3.5. Tumor Explants and Histocultures

Another type of 3D cell model is patient-derived tumor explant (PDE) cultures, in which a tissue fragment is kept alive under ex vivo culture conditions. Explants require minimal manipulation to obtain an in vitro tumor model: the tissue, after mechanical grinding (usually to fragments of 1–3 mm^3^ in size), is placed in a culture medium. This approach allows preserving the heterogeneous composition of the original tumors, including the extracellular matrix and tumor-associated cells [129]. Explants are often used to obtain adherent 2D primary cell cultures, but in the case of the PDE model this is not done, so researchers cultivate explants under special conditions that exclude the migration of plastic-adherent cells from the tissue fragment. To do this, HNSCC explants are placed on an additional supporting matrix, for example, a collagen sponge [20] or a dermal equivalent consisting of human fibroblasts cultured on a viscose fiber fabric [130].

Despite the ease of production and the possibility of preserving the architecture of tumor tissue, the scope of application of PDE as an in vitro model is significantly limited by its fragility; most often, PDE is used for rapid testing of the effectiveness and/or toxicity of drugs within 5–7 days after being obtained [129]. It has been shown that the viability of HNSCC explants decreases from 90% to 30% within a week after the start of cultivation [131]. The survival rate of HNSCC explants can be increased several times by modifying the culture protocol, for example, adding hydrocortisone, aprotinin, ascorbic acid, EGF, or folic acid to the medium [130,131], or placing the explant in more physiological conditions that imitate contact with normal tissue, for example, onto the surface of the dermal equivalent [130] or into cell sheet composing of epithelium and subepithelial stroma [132]. Under such conditions, explants can be maintained in culture for 21 [130] or even more than 30 days [132], while cancer cell heterogeneity and the microenvironment, including vital immune cells, as well as tissue foci of hypoxia, are maintained.

Another option for this type of 3D model is histoculture. When preparing it, tumor tissue, as when obtaining explants, is crushed mechanically, not with surgical instruments, but with the help of a vibratome, which makes it possible to obtain thick sections of unfixed tissue. In these sections, as in explants, tumor cells are retained in their original microenvironment, including the extracellular matrix and immune and stromal cells [133]. The main problem with culturing histocultures is rapid deterioration of tissue condition and loss of cell viability (from 2 [134] to 6 days [135]). However, this time is sufficient to assess the response of tumor tissue to the action of, for example, cisplatin, docetaxel, and cetuximab, while apoptotic fragmentation, activation of caspase 3, and cell loss were observed in treated tumor slices, which demonstrated a heterogeneous individual response [134,135].

Thus, explants and histocultures of HNSCC are easy to obtain and effective for quickly assessing the response of tumor tissue to drug therapy, which suggests their promise for use in personalized medicine.

### 3.6. Microfluidic Devices (Tumor-on-Chip)

At the very beginning, we will make an important note: the cultivation of tumor cells in microfluidic devices is not a separate type of model; rather, it is a special technology for culturing the models described above, providing continuous perfusion and removal of waste from cells and mimicking the function of the circulatory system. Microfluidic devices exploit the physical and chemical properties of liquids and gases at a microscale; in such systems, liquids circulate through channels with dimensions of tens to hundreds of micrometers [136]. Thanks to their small size, microfluidics allow the analysis and use of lower volumes of samples, chemicals, and reagents, reducing the global fees of applications. In addition, this technology makes it possible to very accurately control the impact on the object under study; in the future, it will also make it possible to automate routine research work. To assess the tumor response to exposure, it is possible to analyze both the system effluent (sampling can be carried out at any selected interval, for example, every 2 h) and the object itself after extraction [41,136].

When modeling HNSCC, microfluidic devices are most often used for culturing tumor tissue fragments (explants and histocultures) (Table 4).

Thus, tumor-on-chip technology is a valuable tool for personalized assessment of the effectiveness of various antitumor agents (alone or in combination), allowing dynamic monitoring of the response of tissue samples from patients with HNSCC to treatment. The development of microfluidic platforms can improve patient outcomes through the selection of an optimal personalized treatment strategy.

## 4. In Vitro Cell Models of HNSCC: Which to Choose?

**Figure 1:**
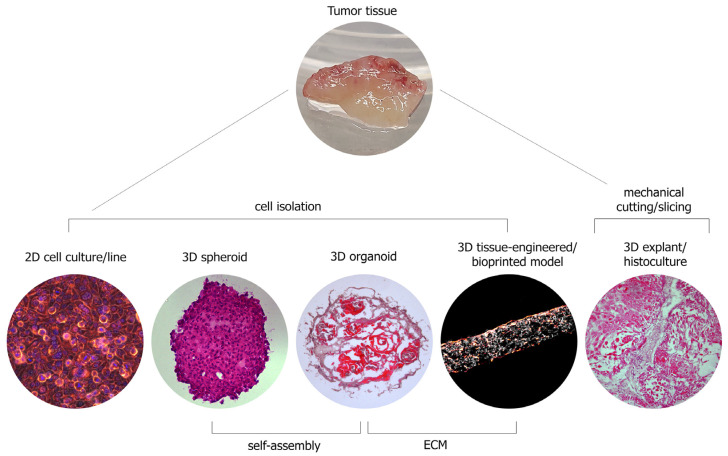
In vitro models of HNSCC.

To study the HNSCC biology, several types of in vitro models, differing in many parameters, are currently actively used (Table 5, Figure 1).

The choice of a specific model depends on the tasks and capabilities of the scientific laboratory. As follows from the bibliometric analysis of research papers on various topics in the PubMed database, the number of investigations in the area of 3D cancer models in vitro is growing every year. However, in 2001–2020, only 65 papers for all types of HNSCC 3D in vitro models were published, which is only 0.72% of all studies involving 3D cancer models. Such a small number of works (HNSCC claims the lives of 450 thousand people a year! [5]) is primarily due to the fact that obtaining a reproducible and relevant 3D model of HNSCC is in itself a rather complex scientific task.

Obviously, 3D models of HNSCC are more valuable from a researcher’s point of view than cell lines or cultures, since they more accurately reproduce the cellular composition and architecture of tumor tissue. These properties allow the use of 3D models to study disease pathogenesis and progression, for which cell–cell interactions and various pathophysiological gradients are important [142,143,144]. Despite varying success rates in generating 3D models, there is consensus regarding their promising potential for testing anticancer agents and enhancing the predictive value of preclinical studies [43,67,119,120,145].

As soon as researchers obtained 3D models for their arsenal, there appeared a need for their direct comparison with 2D lines or primary cell cultures in many parameters, i.e., cell morphology, expression of genes involved in carcinogenesis, production of signaling proteins, and response to drug and radiation therapy [146,147,148,149,150,151]. In the vast majority of such works, the authors concluded that 3D models have an undoubted advantage. Below are a few examples. Schmidt et al. examined the ability of 12 HNSCC cell lines to form spheroids in ultra-low attachment plates, showing that the formation of tight regular spheroids was dependent on distinct E-cadherin expression levels in monolayer cultures. After that, microarray analysis was used to create a gene expression array profile of HNSCC cells, growing as a monolayer and as a 3D spheroid. A global upregulation of gene expression was related to genes involved in cell adhesion, cell junctions, and cytochrome P450-mediated metabolism of xenobiotics, whereas downregulation was associated with genes controlling the cell cycle, DNA-replication, and DNA mismatch repair [152]. In a similar study, Melissaridou et al. compared the properties of five HNSCC lines; when cells transitioned from 2D to 3D culture conditions, there was an increase in the expression of NANOG and SOX2, used as markers of tumor stem cells (for example, for the laryngeal carcinoma line LK1122, expression increased by 26.8 and 22.9 times, respectively), but this did not change the other marker, CD44. The MTS-based assay revealed that cells grown in 3D tumor spheroids showed higher viability after treatment with increasing doses of cisplatin and cetuximab [146]. The increase in drug resistance when moving from 2D to 3D models has been confirmed by numerous scientific groups [43,133]. Typically, we are talking about a 2–20-fold increase in resistance, for example, Cal33 monolayers were 6-, 20-, 10-, and 16-fold more sensitive than spheroids to growth inhibition by ellipticine, idarubicin, daunorubicin, and doxorubicin, respectively [153]. However, in some studies these numbers are even higher: for a bioprinted 3D model, a 4-fold increase in the IC50 of cisplatin and an 80-fold increase for 5-fluorouracil compared to monolayer HNSCC cultures was shown [103]. Monitoring treated 3D models allows the observation of the dynamics of drug penetration and distribution gradients, as well as the identification of markers for drug-resistant cell populations that could represent a source of drug failure and recurrence [147,153].

## 5. In Vitro Cell Models of HNSCC and Oncoviruses

The primary risk factors commonly linked to head and neck cancer encompass tobacco, alcohol consumption, using areca nut, and viral infection [5]. This section considers the development of in vitro models of HNSCC with oncogenic viral infections including human herpes viruses and human papillomaviruses, which play an important role in the pathogenesis of cancer [5,154].

### 5.1. Human Herpes Viruses (HHVs)

Epstein–Barr virus (EBV), also known as human herpes virus type 4 (HHV4), has tropism for B cells and epithelial cells and is closely associated with nasopharynx and oral cavity tumors [154]. Nasopharyngeal and laryngeal tumor incidence are more associated with the EBV in East Asian populations than with human papillomavirus [5]. For example, examination of patients with nonkeratinizing nasopharyngeal carcinomas in southern China and Southeast Asia found 100% of them were EBV-positive [116,155]. In a study by Wang et al. using the EBV-encoded small RNA in situ hybridization assay, 100% of tumor tissue samples and 100% of organoids obtained from patients with nasopharyngeal carcinoma were EBV-positive [116].

Herpes simplex virus 1 (HSV-1) has been implicated in several diseases of varying severity, such as chronic tonsillitis and HNSCC. The HSV-1-positive cases in HNSCC have been associated with the advanced stage (T3/T4) [156]. Driehuis et al. monitored the process of infection of patient-derived oral mucosa organoids using tdTomato-labeled HSV-1; it took 2 weeks for the virus to spread throughout the organoids unless inhibited with acyclovir (a viral tyrosine kinase inhibitor) [123].

### 5.2. Human Papillomavirus (HPV)

More than 200 papillomavirus types infect humans. According to the World Health Organization International Agency for Research on Cancer (WHO IARC), HPV types 16 and 18 are classified as carcinogenic to humans (Group 1), HPV types 31 and 33 are probably carcinogenic (Group 2A), and HPV types 35, 39, 45, 51, 52, 56, 58, and 59 are possibly carcinogenic (Group 2B) [154]. Some researchers suggest considering HPV+ and HPV− HNSCC as separate groups of neoplasms. Compared to HPV-negative, HPV+ HNSCC more commonly affects the oropharynx (predominantly), hypopharynx, and larynx than the oral cavity. Moreover, HPV+ HNSCCs have different pathological patterns, immune signature, and mutation burden, i.e., greater infiltration of B cells into the tumor microenvironment, fewer genetic mutations, and intact apoptotic response, which may explain the improved prognosis and superior response to radio- and immunotherapy and significantly longer median survival than HPV-negative HNSCC (130 months vs. 20 months) [5].

Thus, it is not surprising that when modeling HNSCC in vitro, scientists always emphasize the HPV status of the cell line or tumor cells within the 3D model. HPV accounts for 72% of all head and neck squamous cell carcinoma cases in developed nations [5]; however, in fact, researchers rarely work with HPV+ HNSCC in vitro models. Just compare: the number of currently available HPV+ immortalized HNSCC cell lines is significantly limited, with eight lines known precisely (UMSCC-47, UMSCC-104, UPCI:SCC090, UPCI:SCC152, UPCI:SCC154, 93-VU-147T, HMS001, and LU-HNSCC-26) [20,120,157], while the number of HPV-negative lines is in the hundreds.

The development of primary cell lines from a naturally infected HPV+ cancer is rarely successful [21] due to the low mutational load; for example, the TP53 gene is almost always wild-type, both in tumor tissue and in primary cell cultures or immortalized cell lines derived from it [120,157]. The preservation of genes involved in cell cycle control and apoptosis induction leads to low proliferation rates of HPV+ tumor cells when transferred to a 2D monolayer; the cultures are highly radiation and drug-sensitive and readily enter senescence. It is important to note that immortalized HPV+ cell lines retain the radiation sensitivity both in 2D form and when cultured in the form of spheroids or cell line-derived xenografts [158,159]. Interestingly, some HPV+ immortalized lines are unable to form stable 3D spheroids (for example, UMSCC-47) or only form slowly growing spheroids (for example, UPCI:SCC090) [159].

Similar difficulties are observed with patient-derived 3D models. For a long period of time, the attempts to obtain organoids from HPV-associated HNSCC were unsuccessful. This was primarily for clinical reasons, i.e., the patients with HPV+ HNSCC less commonly receive surgery. One of the first organoids from HNSCC specimens was obtained by Tanaka et al., with the efficiencies similar for HPV+ (3/9) and for HPV− (10/34) patients [120]. In another study, the efficiency of obtaining organoids was higher: eight lines of HPV+ organoids were isolated from nine samples of initially infected tumor tissue [160]. Another approach for obtaining organoids from HPV+ tumor tissue involves the implantation of tumor fragments in NOD/SCID/IL-2Rγ−/− mice to a <25% stable engraftment rate; after the xenograft reaches a volume of 1 cm^3^, it was passaged at least twice, removed, dissociated into individual cells, and cultured on ECM to form organoids. The approach originally yielded a panel of nine HPV+ organoids from nine xenografts [161]; in our opinion, it is overly complicated, and, in addition, the organoids may essentially model not the tumor tissue, but the in vivo model (xenograft).

HPV+ organoids can also be obtained by infection of oral mucosa organoids. Driehuis et al. used HPV16 particles, which led to accumulation of viral DNA in organoids, and after 12 days the presence of virions was confirmed in a conditioned medium [119]. Such models are undoubtedly easier to obtain and suitable for studying the pathogenesis of HPV-associated HNSCC.

Another available HPV+ in vitro model involves patient-derived explants; culturing on a supporting matrix (dermal equivalent) allows maintaining the viability of a tumor tissue fragment for up to 14–21 days depending on the sample. In this model, infection with the virus, confirmed by detection of HPV DNA or IHC staining for the p16INK4a marker, persists even after irradiation [130].

## 6. New Trends in In Vitro Modeling of HNSCC

The development and implementation of a personalized approach in clinical oncology requires constant improvement of preclinical models aimed at maximizing the approximation of cell models to the structure of the native tumor and increasing the efficiency of obtaining such models. For in vitro models of HNSCC, several areas of research can be distinguished.

### 6.1. Models of Vascularization 

In the tumor in vivo microenvironment, there is an active interaction between tumor and endothelial cells, which is important for the recruitment of angiogenic cells, tumor cell survival, and migration [123,162]. In most cases, the method of co-cultivation of 2D cultures is used to analyze the interaction of tumor and endothelial cells [163]. It has been shown that exosomes produced by PCI-13 (HPV−) and UMSCC47 (HPV+) cell lines, as well as exosomes from plasma of HNSCC patients, stimulated proliferation, migration, and tube formation by human umbilical vein endothelial cells (HUVECs) in vitro and promoted formation of defined vascular structures in vivo [164]. To improve inter- and intratumoral vasculature, tumor cells actively produce proangiogenic factors. When 2D HNSCC cell lines are transferred to 3D culture conditions, their proangiogenic potential increases; transplantation of a suspension of primary cancer-associated fibroblasts and 3D spheroids from FaDu cells led to the growth of a well-vascularized tumor in a mouse model of xenografts, while, when transplanting cell suspensions from 2D cultures, gradual necrosis of the graft due to insufficient blood supply to the tissue was observed [165]. To our surprise, we found a description of only one vascularized 3D model of HNSCC. Bessho et al. created a tissue-engineered model of oral cancer; human oral squamous cell carcinoma (HSC-4) cells, human umbilical vein endothelial cells (HUVECs), and normal human dermal fibroblasts were successfully cocultured within gelatin-based matrix, resulting in structures that mimicked 3D-cancer tissues. This model was used to assess sensitivity to X-ray irradiation [166].

Angiogenesis in solid tumor tissue is a promising target for HNSCC-targeted therapy. Researchers are actively searching for signaling molecules and pathways of interaction between epithelial and tumor cells, and tumor-associated lymphocytes (including B cells and T cells) [167] or macrophages [43] can act as mediators in this interaction. To create a relevant model that is as close as possible to native tissue, it is necessary to reproduce in vitro one of the key processes of tumor progression and metastasis-angiogenesis [168,169]. The furthest along this path has been bioprinting technology, which can directly bioprint the vascular wall structures or create hollow channels inside a volumetric matrix that will later be populated by endothelial cells [162]. Microfluidic technologies also make it possible to study various aspects of the interaction between tumor and endothelial cells in vitro: metastasis (intra- and extravasation), chemotaxis, production of angiogenic factors, etc. [170,171]. New prospects, in our opinion, are also opening up for patient-derived explant cultures: culturing an explant of tumor tissue on an artificial vascular bed (co-culturing vascular endothelial cells and smooth muscle cells in a matrix comprising ECM components and growth factors) can increase the duration of its existence ex vivo [129].

### 6.2. New Types of Matrices for Tumor Cell Culturing

Currently, there is an active process of abandoning xenogeneic reagents when modeling human tissues in vitro in favor of autogenous or allogeneic materials; it is assumed that such a replacement will make it possible to recreate a more favorable microenvironment for cells, simulating their original environment in vivo [172]. Thus, when modeling HNSCC in vitro, it was proposed to use allogeneic gels based on human tumor tissues—human uterine leiomyoma-derived Myogel or human pre-metastatic neck lymph node-derived Lymphogel, instead of Matrigel (solubilized basement membrane preparation extracted from the Engelbreth-Holm-Swarm mouse sarcoma) [173,174]. Such matrices differ in their protein composition, including the content of unique adhesion-related proteins [174]. An experiment involving 12 HNSCC cell lines revealed an increase in the resistance of tumor cells to the action of EGFR and MEK inhibitors compared to cells cultured on plastic or in Matrigel. The authors of the work suggest that the human tumor matrix improves the predictability of in vitro anticancer drug testing [173].

## 7. Three-Dimensional In Vitro Cell Models of HNSCC for Personalized Medicine

Currently, the attention of researchers is aimed at the development of personalized medicine, where an important tool is 3D models obtained from tumor tissue of patients. The main goal is to obtain a model that can predict the patient’s response to therapy in order to optimize the treatment strategy [175]. In the case of HNSCC, we can already talk about certain successes in this direction.

Valuable information for clinicians can be obtained by using patient-derived explants (including those cultured using microfluidic devices) to test antitumor therapy [176]. Such studies already make it possible to identify inter- and intra-patient variability in response to irradiation and chemotherapy [138].

Tanaka et al. [120] managed to obtain HNSCC organoids that were as close as possible to the primary tumor; they had a similar morphology, and retained the expression of markers of mesenchymal (vimentin) and tumor (CD44 and ALDH1A1) cells, although CD68+ cells were found only in the tumor, but not in the organoids. When testing sensitivity to platinum and docetaxel therapy in the resulting organoids, consistency was found between the response in vitro and in vivo [120].

Driehuis et al., analyzing the expression of markers TP63, TP40, Ki67, CK13, and CK5, convincingly showed that organoids obtained from the mucous membrane of the oral cavity or tongue recapitulate the functional and morphological characteristics of the tumor [119,177]. The same study found that the response of organoids to radiation correlated with the response of patients; three organoid lines were among the most resistant when exposed to radiotherapy in vitro, and three corresponding patients relapsed after undergoing radiotherapy; moreover, a patient whose organoid line showed the highest sensitivity to irradiation had a lasting response to palliative radiotherapy [119].

Millen et al. describes the organoid biobank, which stores a collection of biomaterial from patients with HNSCC from various anatomical locations and histological cancer subtypes. During routine surgical resection or biopsy procedures in 2019–2022, samples were collected from 228 patients, but organoids were successfully obtained from only 97 patients (42.5% efficiency). To store organoids, the biobank uses cryopreservation technology; the proportion of successfully thawed organoids was 70.9%. The resulting organoids retained histopathological and molecular features of primary tumor tissue; tumor cells in their composition expressed CK13 and p63, and a genetic study revealed mutations in the tumor-associated genes TP53 (in 63% of organoids), NOTCH1, PIK3CA, FAT1, and APOB, gains of oncogenes including PIK3CA, FGF3, and FGF4, and loss of tumor suppressor CDKN2A. The collection included unique samples, for example, four salivary gland tumor organoid models that retained the ability to produce mucin and amylase, as well as organoids from tissues of a patient with Fanconi anemia, which had increased sensitivity to double-stranded DNA breaks induced by mitomycin-C. Despite the similarity of organoids and tumor tissue at the cellular and molecular levels, in combined treatment (radiation therapy + cisplatin) no clear correlation between organoid and patient response was observed. At the same time, the organoid response to radiotherapy (measured as organoid viability at 2 Gy) correlated with clinical relapse status in the adjuvant setting but not in the primary setting. In addition, the researchers successfully used CRISPR-Cas9 base-editing technology to introduce the E545K mutation (one of the most common PIK3CA mutations), which opens up new opportunities for scientists to use patient-derived 3D models to search for and validate potential biomarkers of HNSCC response to therapy [160].

Promising results of experimental work allowed scientists to move on to promoting clinical trials using in vitro models of HNSCC. In October 2023, only three clinical trials were registered, which is entirely attributable to the difficulty of obtaining patient-derived models of head and neck cancer.

The ORGAVADS (ORGAnoids + VADS (French “voies aérodigestives supérieures”) tumors) study is a multicenter observational trial (NCT04261192) conducted to investigate the feasibility of generating and testing patient-derived tumor organoids derived from HNSCC for the evaluation of sensitivity to treatments (chemotherapy, radiotherapy, PARP inhibitors, and immunotherapy). The planned number of study participants is determined taking into account that the expected efficiency of obtaining organoids from patient material will be about 60%. The sponsor (Centre Francois Baclesse) assumes that this screening could make it possible to refine the choice of treatments adapted to each patient and thus limit the undesirable effects [178,179].

Investigators hypothesize that high-throughput screening on patient-derived tumor organoids can be used as an adjunct tool to aid treatment selection in patients with cancer. The objective of the NCT04279509 study is to determine if a drug screen assay (panel of ten primary and five additional anticancer drugs) using personal organoid models can accurately select a chemotherapeutic agent that results in objective response in patients with refractory solid tumors (including HNSCC) [180].

The SOTO (Sensitivity of Organoids to Treatment Outcome) prospective observational study (NCT05400239) aims to determine the sensitivity of organoids obtained from patients with HNSCC to radiotherapy, platinum (cisplatin and/or carboplatin) chemotherapy, or cetuximab or their combination, with subsequent determination of the correlation of the treatment sensitivities of organoids with the treatment outcome of patients [181].

If successful, the clinical trials described above will mark a new stage in the development of personalized medicine in head and neck cancer.

## 8. Conclusions

For several decades, researchers have been trying to answer the demand of clinical oncologists to create an ideal preclinical model of HNSCC that is accessible, reproducible, and relevant. Over the past years, the development of cellular technologies has naturally allowed us to move from short-lived primary 2D cell cultures to complex patient-derived 3D models that reproduce the cellular composition, architecture, mutation, or viral load of native tumor tissue. The variety of models developed in vitro allows scientific teams to solve a wide variety of problems: screening agents with potential antitumor activity, studying the contribution of the tumor microenvironment to its progression and metastasis, determining the prognostic significance of individual biomarkers (including using genetic engineering methods), studying the influence of viral infection on the pathogenesis of the disease, and adjusting the treatment tactics for a specific patient or groups of patients. Promising experimental results have created a scientific basis for the registration of several clinical studies using in vitro models of HNSCC. It can be assumed that in the coming years, the field of in vitro modeling of tumor tissues will actively develop: biobanks of such models will become widespread, new biomarkers of HNSCC with high predictive value will be identified, and the proportion of successful clinical trials and the effectiveness of treatment will increase due to more accurate results of preclinical studies.

## Figures and Tables

**Table 1:** The Head and Neck Cancer Cell Lines Panel.

Cell Line	Tissue	Tumor Source	Sex	Karyotype	Mutant Genes
A-253	submaxillary salivary gland	primary	male	near triploid with at least 6 markers	*CDKN2A* *KDMC5* *TP53*
SCC-9	Tongue	primary	male	n.d.	*CDKN2A* *TP53*
SCC-15	Tongue	primary	male	n.d.	*TP53*
SCC-25	Tongue	primary	male	possible loss of Y chromosome	*CDKN2A* *TP53*
FaDu	hypopharynx	primary	male	hypodiploid to hypertriploid with modal number = 64	*CDKN2A* *SMAD4* *TP53*
Detroit 562	Pharynx	metastasis (pleural effusion)	female	modal number = 64; range = 58 to 128	*CDKN2A* *PIK3CA* *TP53*

**Table 2:** Examples of research using heterospheroids to study HNSCC.

HNSCC Cell Line (Organ)	Additional Cellular Component	Ratio	Research Tasks	Ref.
FaDu (pharynx)	MeWo (granular fibroblasts, derived from human melanoma)	5:1	To study the effect of stromal components on delivery of nanoparticles into the tumors	[74]
FaDu (pharynx)	MeWo (granular fibroblasts, derived from human melanoma)	from 10:1 to 1:2	To study penetration, distribution, and antitumor efficacy of photoactive drugs	[75]
UM-SCC-1 (floor of mouth)	NHLF (human lung fibroblasts)	1:1	To study the application of high-density lipoprotein nanoparticle as a biocompatible delivery system for a well-established radio-sensitizing miR-34a	[76]
LK0902 (tongue), LK0917 (gingiva), or LK1108 (hypopharynx)	CAF (cancer-associated fibroblasts)	from 2:1 to 3:1	To investigate the impact of CAFs on phenotype, proliferation and cisplatin and cetuximab treatment response in HNSCC cells	[77]

**Table 3:** Culture media and production efficiency of HNSCC organoids.

	Perréard, 2023 [112]	Wang, 2022 [116]	Driehuis, 2020 [99]	Kijima, 2019 [117]	Zhao, 2019 [48]	Tanaka, 2018 [29]
	**Culture Media**					
Basal media	adDMEM/F12	n.d.	adDMEM/F12	adDMEM/F12	DMEM/F12	StemPro hESC
Penicillin-streptomycin	100 U/mL		100 U/mL	100 U/mL		
Primocin	100 μg/mL					
HEPES			10 mM	10 mM		
GlutaMAX	1×		1×	1×		
B27 supplement	1×		1×	1×	1×	
N2 supplement				1×	1×	
N-acetyl-L-cysteine	1.25 mM		1.25 mM	0.1 mM		
Nicotinamide	10 mM		10 mM	10 nM		
hEGF	50 ng/mL	5 ng/mL	50 ng/mL	50 ng/mL	50 ng/mL	
hFGF-10	10 ng/mL		10 ng/mL			
hFGF-2	5 ng/mL	5 ng/mL	5 ng/mL			8 ng/mL
A83-01	500 nM		500 nM	500 nM	500 nM	
Prostaglandin E2	1 μM		1 µM			
CHIR-99021	0.3 μM		0.3 µM			
Forskolin	1 μM		1 µM			
Gastrin				10 nM	10 nM	
Y-27632	10 μM	10 ng/mL		10 μM		
Wnt3A		250 ng/mL		100 ng/mL		
SB202190				10 nM		
R-spondin-1		500 ng/mL				
Noggin		500 ng/mL				
R-spondin-1-conditioned media	10%					
Wnt3a, R-spondin-3, Noggin-conditioned media	50%					
R-spondin-3-Fc fusion protein conditioned medium			4% (*v*/*v*)			
Noggin-Fc fusion protein conditioned medium			4% (*v*/*v*)			
Noggin/R-Spondin conditioned media				2% (*v*/*v*)		
	Overall efficacy of organoid generation					
	assumed around 60%	62.9% (39/62)	around 70%	80% (4/5)	n.d.	30.2% (13/43)

**Table 4:** Examples of research using microfluidic devices to study HNSCC.

Object	In Vitro Culture Duration	Exposure	Analysis of System Effluent	Analysis of the Object	Ref.
HNSCC biopsies (5–10 mg)	2 days	-	-	Morphology (H&E staining), cell death (flow cytometry after PI staining), cell viability (MTS proliferation assay)	[136]
HNSCC biopsies (5–10 mg)	6 days	Irradiation (2–40 Gy)	Cell death (detection of LDH and cytochrome c release)	Apoptosis (IHC for caspase-cleaved CK18)	[137]
HNSCC biopsies (5–10 mg)	2 days	Irradiation (5–20 Gy)	Cell death (detection of LDH release)	Apoptosis (IHC for caspase-cleaved CK18), DNA damage (IHC for phosphorylated-H2AX, TUNEL assay), cell proliferation (IHC for Ki67)	[138]
HNSCC slices (discs 5 × 0.35 mm)	68 h	Irradiation (5 × 2 Gy), chemotherapy agent (cisplatin)	Cell death (detection of LDH release)	Morphology (H&E staining), apoptosis (IHC for caspase-cleaved CK18), DNA damage (IHC for phosphorylated-H2AX), cell proliferation (IHC for Ki67 and BrdU)	[139]
HNSCC biopsies (5–10 mg)	9 days	Chemotherapy agents (cisplatin, 5-flurouracil, docetaxel)	Cell death (detection of LDH release), cell viability (WST-1 proliferation assay)	-	[140]
HNSCC biopsies (5–10 mg)	7 days	Chemotherapy agents (cisplatin, 5-flurouracil)	Cell death (detection of LDH and cytochrome c release), cell viability (WST-1 proliferation assay)	Morphology (H&E staining)	[141]

**Table 5:** In vitro models of HNSCC: main features.

	2D		3D				
	Immortalized Cell Lines	Primary Cell Cultures	Spheroids and Heterospheroids	Tissue-Engineered Models	Bioprinted Models	Organoids	Explants and Histocultures
Source	cell biobanks	patient-derived tissue	patient-derived tissue, primary cell cultures, immortalized cell lines			patient-derived tissue	
Heterogeneity of tumor cellular composition	not preserved	partially preserved	depends on the source			preserved	
ECM	no			natural and synthetic polymers, decellularized tissue	bioink based on hydrogels	basement membrane matrix, collagen	native
Tissue architecture, pathophysiological gradients	absent		partially reconstituted	reconstituted			preserved
In vitro culture duration	not limited	Limited					
Difficulty of obtaining	low	medium			high		Medium
Major advantages	availability, stability of properties, many years of experience in use, ability to obtain a 3D model	availability, ability to obtain a 3D model	the most available 3D model	convenience of studying the ECM–cells interaction, possibility of getting a model with given linear dimensions	obtaining artificial tumor tissue with specified spatial characteristics	capability to support tumor cells at different levels of differentiation, mimicking the tumor microenvironment	minimally manipulated tumor tissue
Specific disadvantages	chromosomal instability, impossibility of use for personalized medicine	the initial ratio of tumor and tumor-associated cells and their properties may change during cultivation	prone to fusion to form conglomerates, difficulty in controlling size	a lot of cells are required for modeling	a lot of cells are required for modeling, sophisticated equipment is required	production efficiency about 60–70%	long-term in vitro cultivation requires supporting matrices or microfluidic devices

## Data Availability

Not applicable.
